# Formal and informal human milk donation in New Zealand: a mixed-method national survey

**DOI:** 10.1186/s13006-024-00667-4

**Published:** 2024-09-02

**Authors:** Shalee Harris, Frank H. Bloomfield, Mariana Muelbert

**Affiliations:** Liggins Institute, University of Auckland, Auckland, New Zealand

**Keywords:** Infant, Nutrition, Human milk, Donation, Human milk bank, Informal milk donation, Milk sharing

## Abstract

**Background:**

Mother’s milk provides optimal nutrition for infants. Donor human milk (DHM) is recommended for low birthweight infants when mother’s milk is unavailable. Little is known about human milk (HM) donation practices in New Zealand (NZ), where few HM banks are available. This study aimed to investigate parents’ and health professionals’ (HP) experiences with formal and informal HM donation in NZ.

**Methods:**

Two electronic surveys were disseminated in 2022 to parents and HPs involved with HM donation in NZ. The surveys covered respondents’ views and experiences with HM donation. HPs were also asked about HM donation practices in their workplace. Chi-squared and Fisher-Freeman-Halton exact tests were used for quantitative analysis and qualitative data were thematically analysed using inductive approach.

**Results:**

A total of 232 HP and 496 parents completed the surveys. Most parents either donated (52%) or sought DHM (26%) for their infant and most donations were informal, arranged between individuals (52%) or through hospital staff (22%). HP reported DHM was used in 86% of facilities, with only 20% of donations facilitated by HM banks. Almost half (48%) of HP stated they would like to use DHM in their workplace but access was limited. The most common screening processes undertaken by parents and HP before informal HM donation were lifestyle including smoking status, medication, drug and alcohol intake (44% and 36%, respectively) and serological screening such as CMV, HIV, Hepatitis C or B (30% and 39%, respectively). Pasteurisation of DHM obtained informally was not common. Most donors were satisfied with their HM donation experiences (informal and/or formal, 91%) and most respondents supported use of DHM in hospitals and community. Participants reported HM donation could be improved (e.g., better access) and identified potential benefits (e.g., species-specific nutrition) and risks (e.g., pathogens) for the infant. Potential benefits for the donor were also identified (e.g., altruism), but respondents acknowledged potential negative impacts (e.g., cost).

**Conclusion:**

Informal HM donation in NZ is common. Most parents and HP support the use of DHM; however, improvements to current practices are needed to ensure safer and more equitable access to DHM.

**Supplementary Information:**

The online version contains supplementary material available at 10.1186/s13006-024-00667-4.

## Background

Mother’s milk is recognised as the preferred source of nutrition for an infant [1]. This is largely due to maternal milk providing key components for optimal infant growth and development beyond nutrients. Such beneficial properties include growth factors, metabolic hormones, and a wide variety of immunological and biologically active components [2–5]. However, many factors can prevent a baby from receiving their mother’s milk, including delayed or insufficient milk production, prolonged periods of maternal and infant separation, inability to suckle and illness [6–8]. In circumstances where mother’s milk is not available or insufficient, alternative feeding methods are required. For very low birthweight and very preterm infants, the World Health Organization and UNICEF recommend donor human milk (DHM) as the best alternative [9].

Approximately 5–18% of infants worldwide are born preterm and rates continue to increase [10]. Due to immature coordination of the suck-swallow-breathe reflexes and limited availability of mothers’ milk in the first days after preterm birth, clinicians must decide the best nutritional approach while waiting for maternal milk supply to meet demand [11, 12].

The use of DHM in very low birthweight and very preterm infants instead of infant formula is associated with a multitude of health benefits, such as reduced risk of necrotising enterocolitis, improved feeding tolerance and increased rates of exclusive breastfeeding [13–16]. With growing recognition of the clinical benefits of DHM, interest in human milk (HM) banks and informal milk sharing have proliferated globally [17].

In New Zealand (NZ), however, provision of DHM is difficult due to limited availability of HM banks. Currently, there are only six active HM banks (all non-profit), none of which are in Auckland (the largest, most populated city of NZ with two tertiary neonatal intensive care units). As consequence, informal milk sharing (also known as peer-to-peer donation) is a popular alternative for parents seeking to donate or receive DHM to avoid unnecessary exposure to infant formula [18], which has been reported to be associated with long-term health effects such as overweight/obesity and adverse cardiometabolic outcomes [19–21]. Informal milk sharing is not facilitated by a HM bank; instead, it involves using social networks, including friends, family, social media, or parents of preterm infants within the same neonatal unit to exchange expressed HM for the purpose of infant feeding [18, 22]. However, feeding infants raw unscreened HM can result in inadvertent transfer of pathogens through milk and expose the infant to severe illnesses, including Human Immunodeficiency Virus and Cytomegalovirus (CMV) [23], which may go unrecognised without specific testing or HM pasteurisation. Furthermore, informal milk sharing is open to a myriad of collection, storage and transportation techniques that can create conditions for pathogenic bacteria to grow, which may further increase the risk of microbiological contamination [24].

Anecdotal information suggests informal milk sharing in NZ often occurs via individual arrangements between parents [25]; however, no formal research has been conducted. Thus, the aim of this research was to investigate parents’ and health professionals’ (HP) perceptions and experiences with HM donation in NZ, both within hospitals and in the community.

## Methods

### Design

Two electronic mixed-method surveys were created, one for parents and one for HP, containing open- (free text) and closed- (multiple choice) questions (Additional file 1). The surveys included a range of questions regarding parents’ and HP demographic information, experiences and perceptions regarding both formal and informal HM donation practices. Questions were created in consultation with stakeholders, consumers and cultural advisors to capture experiences relevant to the NZ population. In addition, prior to going live both surveys were tested with a small group of parents and health professionals to ensure adequate readability and length of survey.

### Sampling and recruitment

The surveys were circulated throughout New Zealand from 1^st^ of April to 1^st^ of July 2022, simultaneously via email to key stakeholders (health professional organisations involved in maternity or neonatal care, such as Perinatal Society of Australia and New Zealand, Paediatric Society of New Zealand, New Zealand College of Midwives, New Zealand Breastfeeding Alliance, Australasian Neonatal Dietitians Network) and via advertisements on social media platforms (Facebook, Twitter and LinkedIn). A snowball dissemination strategy was used, in which participants were asked to share the survey among their networks. Social media advertising strategies were also utilised to target specific populations (HP, parents with young infants and NZ native Māori participants). As there was no estimation of how many HP and parents had been involved with milk donation in New Zealand, no formal sample size calculation was undertaken.

### Eligibility criteria

HP were invited to participate in the survey if they, in the last five years, were involved in the facilitation of formal (via HM bank) or informal HM donation in NZ. Involvement with HM donation was defined as being an HP (neonatologist, dietitian, lactation consultant, midwife, neonatal nurse, or other qualified HP) who facilitated the donation and/or receipt of DHM, or who was directly involved in the neonatal care of an infant receiving DHM.

Parents of infants born after 1^st^ January 2018 and who had been directly involved with formal (via HM bank) or informal HM donation were invited to participate in the survey. Direct involvement was defined as having either donated their own milk to a HM bank or to another parent, received DHM for their infant from a HM bank or from another parent, or both. Those who were not directly involved, but wished they could have had the opportunity to use DHM, were also invited to participate in the survey.

Surveys were developed in Qualtrics and ethical approval was granted by the Auckland Health Research Ethics Committee (AHREC application #AH23817). Survey responses were anonymous and all participants provided electronic consent to participate in the survey.

### Statistical analysis

Quantitative data (count, frequencies) are presented as frequencies of the total number of responses to each question (%). Respondents were not required to answer all survey questions, and some questions allowed for multiple answers. The final number of responses for each question is shown in each table. Given the use of a snowball technique to disseminate the survey, it is not possible to report a response rate . Chi-squared test and Fisher-Freeman-Halton exact test were used to investigate associations between categorical variables. IBM SPSS Statistics 28 was used for statistical analysis. A p-value below 0.05 was considered statistically significant.

Qualitative data (free text) were thematically analysed in NVivo software using an inductive approach, consisting of identification of common phrases and recurring words by NVivo, which were then validated by the research team and classified into relevant thematic groups, and categorised into relevant overarching themes related to the answers provided by both parents and HP separately.

## Results

### Study population

A total of 566 parents responded to the survey. Seventy responses were incomplete and therefore were excluded from the analysis for the following reasons: declined consent (*n* = 1), no questions answered (*n* = 6) and incomplete responses (*n* = 63). Therefore, a total of 496 responses were included in the final analysis.

A total of 283 HP responded to the survey. Fifty-one responses were incomplete and therefore were excluded from the analysis for the following reasons: unsubmitted (*n* = 39), no questions answered (*n* = 3), declined consent (*n* = 1) and incomplete responses (*n* = 8). Therefore, 232 HP responses were included in the final analysis.

Demographic details of parents, infants and HPs are shown in Table 1. Of the parents who responded to the survey (*n* = 496), the majority were aged between 30 and 39 years (64%) and were of NZ/European descent (76%). Participants of Māori decent (Indigenous people of NZ) constituted 7.6% of respondents and most respondents were from the North Island of NZ (72%). Infants’ age at survey completion was evenly distributed and majority were girls (55%). Most infants were born in hospital (82%) and required postnatal care of some form (59%), most often on the postnatal ward (30%).

**Table 1:** Study population

Parents’ and infants’ demographic characteristics	Number of participants, *n* (%)
Parent Age (*n* = 496)^†^	
<30	108 (21.8)
30–39	317 (63.9)
≥40	70 (14.1)
Undisclosed	1 (0.2)
Parent Ethnicity (*n* = 552)^†^*	
New Zealand European	418 (75.6)
Māori	42 (7.6)
Samoan	3 (0.5)
Cook Islands Māori	1 (0.2)
Chinese	12 (2.2)
Indian	8 (1.3)
Other (e.g., English, European, Asian)	68 (12.3)
Place of birth (*n* = 493)^†^	
Hospital	403 (81.7)
Birthing centre/maternity unit	29 (5.9)
Home birth	54 (11)
Other	7 (1.4)
Level of postnatal care (*n* = 496)^†^	
Postnatal ward	150 (30.2)
Special Care Baby Unit	32 (6.5)
Neonatal Intensive Care Unit	113 (22.8)
No postnatal care was required	182 (36.7)
Other (e.g., birth centre)	19 (3.8)
Infant age (*n* = 496)^†^	
0–6 months	157 (31.7)
7–12 months	71 (14.3)
1–2 years	122 (24.6)
≥2 years	146 (29.4)
Infant sex (n = 496)^†^	
Boy	216 (43.5)
Girl	273 (55.1)
Twins of different sex	4 (0.8)
Undisclosed	3 (0.6)
Location (*n* = 495) ^†^	
North Island of NZ	356 (71.9)
South Island of NZ	139 (28.1)

Health professionals’ demographic characteristics	Number of participants, *n* (%)
Gender (*n* = 230)^†^	
Female	225 (97.8)
Male	3 (1.3)
Undisclosed	2 (0.9)
Age (*n* = 229)^†^	
18–39	81 (35.3)
40–49	52 (22.7)
≥50	91 (39.7)
Undisclosed	5 (2.3)
Ethnicity (*n* = 232)^†^*	
New Zealand European	178 (69.5)
Māori	14 (5.5)
Chinese	2 (0.8)
Indian	3 (1.2)
Other (e.g., English, European, South African)	52 (20.3)
Undisclosed	7 (2.7)
Health profession (*n* = 232)^†^*	
Neonatologist	6 (1.9)
Dietitian	6 (1.9)
Lactation Consultant	46 (14.6)
Midwife	138 (43.7)
Nurse	56 (17.7)
Lead Maternity Carer	26 (8.2)
Paediatrician	6 (1.9)
Other	32 (10.1)
Organisation (*n* = 232)^†^	
Governmental Organisation (District Health Board)**	134 (57.8)
Non-governmental Organisation	10 (4.3)
Plunket	5 (2.2)
Private Care	8 (3.4)
Self-employed	62 (26.7)
Other	13 (5.6)
Level of healthcare (*n* = 229)^†^*	
Primary	94 (32.3)
Secondary	82 (28.2)
Tertiary	76 (26.1)
Other (e.g., community-based work)	39 (13.4)
Location (*n* = 115) ^†^	
North Island of NZ	96 (83.5)
South Island of NZ	19 (16.5)
Years of experience in neonatal health (*n* = 230)^†^	
≤10	80 (34.8)
11–15	33 (14.3)
≥15	117 (50.9)

^†^Response count. *Participants could select multiple answers

**District Health Board = organisations responsible for providing health and disability services within designated regions of New Zealand

Among the HP who responded to the survey (*n* = 232), almost all (98%) were female and of NZ/European descent (69%), aged 40 or above (65%) and with 15 or more years of experience working in neonatal health (51%). Most were midwives (44%) working within a governmental organisation such as a district health board (58%) and practising across a range of primary (32%), secondary (21%) and tertiary (26%) levels of care.

### Involvement with human milk donation

Details of parents’ experiences with HM donation are shown in Table 2. Over half (52%) had donated HM or had received DHM during their infant’s hospital stay (12%) or following discharge from the hospital (13%). Some parents wished they had been involved with HM donation, but it was not available to them (14%, of which 3 in 4 were located in the North Island of NZ). Most donations were informally organised between individuals (family/friends, 52%) or facilitated through hospital staff (22%).

**Table 2:** Feeding experiences reported by parents

Infant feeding experiences	Number of participants, *n* (%)
Involvement with HM donation (*n* = 496)^†^	
HM donor	256 (51.6)
Recipient of DHM during hospital admission	61 (12.3)
Recipient of DHM after discharge from hospital	65 (13.1)
Both donated and received DHM	39 (7.9)
Wished to be involved but it was not available	69 (13.9)
Directly breastfed another mother’s infant	6 (1.2)
HM donation arrangement (*n* = 550)^†^*	
Through the hospital	120 (21.8)
Through a milk bank	88 (16)
Between individuals	285 (51.8)
Through charities/NGOs	54 (9.8)
Other	3 (0.6)
Infant mode of feeding in the first six months of life (*n* = 496)^†^	
Exclusively fed mother’s milk	294 (59.3)
Partially fed mother’s milk and infant formula	56 (11.3)
Partially fed mother’s milk and DHM	86 (17.3)
Partially fed DHM and infant formula	10 (2)
Exclusively fed infant formula	3 (0.6)
Exclusively fed DHM	1 (0.2)
Other	46 (9.3)
Health professional support with breastfeeding (*n* = 494)^†^	
Yes	419 (84.8)
No	75 (15.2)
**HUMAN MILK DONORS**	
Frequency of Donations (*n* = 292)^†^	
Daily	36 (12.3)
Weekly	69 (23.6)
Monthly	62 (21.2)
One-off donation	125 (42.8)
**MOTHERS OF DHM RECIPIENT INFANTS**	
Frequency of DHM use (*n* = 164)^†^	
Multiple times per day	125 (76.2)
Once daily	24 (14.7)
Weekly	3 (1.8)
Fortnightly	-
Less than fortnightly	12 (7.3)

^†^Response count. *Participants could select multiple answers

DHM: donor human milk; HM: human milk; NGO: non-governmental organisations

86% of HP reported that DHM was available in their workplace and HM donations were most often (39%) organised via individual arrangements (family/friends/internet/social media), with only 20% of DHM obtained via HM banks (Table 3). No significant association was found between the availability of DHM and the type of organisation that the HP worked (test statistic [*t*] = 5.2, *p* = 0.13; data not shown). Highest rates of formal HM donation were reported for district health boards with established HM banks (Canterbury: 92%; Midcentral: 78%, and Capital & Coast: 67%). In contrast, HM donation across Waikato (77%), Hawke’s Bay (87%) and Hutt (75%) were most reliant on informal arrangements facilitated by hospital staff.

**Table 3:** Donor human milk availability reported by health professionals

Donor human milk availability	Response count, *n* (%)
Availability of DHM (*n* = 232)^†^*	
Yes – Facilitated through the hospital staff	90 (27.4)
Yes – Via a human milk bank	65 (19.8)
Yes – Organised between individuals	127 (38.7)
No	46 (14)
Maternal consent required for informal milk donation (n = 180)^†^	
Yes	168 (93.3)
No	7 (3.9)
Unsure	5 (2.8)
Frequency of DHM use (*n* = 182)^†^	
Often (e.g., daily/weekly)	69 (37.9)
Sometimes (e.g., fortnightly/monthly)	52 (28.6)
Rarely (e.g., quarterly/annually)	51 (28)
Never	-
Unsure	10 (5.5)
Limited availability of DHM restricting use (*n* = 182)^†^	
Often (e.g., daily/weekly)	87 (47.8)
Sometimes (e.g., fortnightly/monthly)	41 (22.5)
Rarely (e.g., quarterly/annually)	32 (17.6)
Never	4 (2.2)
Unsure	18 (9.9)
Availability of guidelines/procedures relating to the use of informal DHM (*n* = 182)^†^	
Yes	129 (70.9)
No	26 (14.3)
Unsure	27 (14.8)

^†^Response count. *Participants could select multiple answers

DHM: donor human milk

### Donor human milk processing

Details on parental and HP management of DHM are shown in Table 4. Prior to DHM exchanges, parents most commonly undertook lifestyle (i.e., smoking status, medication, drug and alcohol intake, 44%) and serological (i.e., CMV, HIV, Hepatitis C or B 30%) screening processes (Table 4). Parents reported most DHM obtained via informal milk sharing was not pasteurised prior to the DHM exchange (60%). Among those who reported DHM was pasteurised by a human milk bank prior to infant consumption (15%), infants born in the South Island of NZ more frequently received pasteurised milk than infants born in the North Island of NZ (37.2% vs. 11%, *t* = 37.1, *p* < 0.01). Some parents reported home-pasteurisation was undertaken by the donor or recipient parent (3%), and the majority described scalding the milk or using a water bath for various periods of time.

**Table 4:** Management of donor human milk

Management of donor human milk	Number of parents, *n* (%)	Number of HP, *n* (%)
Number of screening processes undertaken ^†^		
1 screening	192 (47.4)	19 (12.7)
2 screenings	158 (39)	77 (51.3)
3 screenings	51 (12.6)	28 (18.7)
Screening processes undertaken prior to DHM exchange ^†^*		
Lifestyle (i.e., smoking, medication, drug and alcohol intake)	294 (43.6)	122 (36)
Serological (i.e., blood test, HIV, CMV, Hepatitis C or B, syphilis)	202 (29.9)	130 (38.6)
Microbiological (i.e., bacterial growth)	52 (7.7)	32 (9.4)
None	99 (14.7)	26 (7.7)
Unsure	28 (4.1)	29 (8.6)
Was the Milk Pasteurised/Flash-heated? ^†^		
Yes – through donor or recipient mother	14 (3.4)	-
Yes – through milk bank or hospital facility	63 (15.2)	49 (27.7)
No	250 (60.4)	100 (56.5)
Unsure	87 (21)	28 (15.8)
Were there any associated costs? ^†^		
Yes	144 (35)	
No	268 (65)	
Who paid the associated costs? ^†^		
Healthcare system	11 (7.6)	68 (38.6)
Donor	54 (37.5)	32 (18.1)
Recipient mother	42 (29.2)	
Charity	9 (6.2)	11 (6.3)
Other	21 (14.6)	47 (26.7)
Unsure	7 (4.9)	18 (10.2)

^†^Response count. *Participants could select multiple answers

DHM = donor human milk

Among HP, the majority reported that serological (38%) and lifestyle (36%) screening were undertaken prior to the distribution of DHM (Table 4). HP working within a governmental organisation (district health boards or community paediatric service) more frequently undertook three screening processes (27%) than those who worked for non-governmental organisations/charities/trusts (14%) and private care/self-employed (6.5%, *t* = 15.5, *p* = 0.04).

Pasteurisation of DHM was reported by almost 30% of HP and was more frequent among those working in the South compared to North Island of NZ (73% vs. 27%, respectively, *X*^*2*^ = 11, p = < 0.01), but did not differ among organisations (*t* = 4.7, *p* = 0.19). Additionally, nutritional composition of DHM was often not analysed (69%).

### Donor human milk utilisation

Most parents reported using DHM multiple times daily to feed their infant (76%), and the duration of DHM use varied for four weeks or longer (42.9%) or for under one week (28.9%). The duration of DHM use was significantly associated with the geographical region in which the infant was born (*t* = 8.6, *p* = 0.03, data not shown), with infants born in the North Island more frequently receiving DHM for four weeks or longer (52%) compared to infants born in the South Island (29.2%). Among donors, donations were often a one-off donation (43%, Table 2).

HP were asked which criteria were used in their workplace for the provision of DHM and could select multiple answers. Most (53%) reported that four or fewer criteria were used to determine which infants received DHM and that DHM was commonly used for full-term (72%), early term (65%) and late preterm (58.2%) infants. There was a significant association between the level of care for which the HP worked and the criteria used to allocate DHM (Table 5), with very low birthweight infants more frequently receiving DHM when being cared for under tertiary or secondary levels of care (*p* = 0.02), and preterm infants when being cared for under secondary level of care (*p* < 0.001).

**Table 5:** Proportion of respondents reporting which categories of infants receive donor human milk under their level of care

Criteria	Primary (*n* = 46)	Secondary (*n* = 38)	Tertiary (*n* = 40)	Other (*n* = 13)	Mix* (*n* = 37)	*P* value**
Preterm infants	19 (41.3%)^a^	30 (78.9%)^b^	30 (32.5%) ^a^	5 (38.5%) ^a^	27 (73%) ^a, b^	< 0.001
Term infants	39 (84.8%) ^a^	32 (84.2%) ^a^	18 (45%) ^b^	11 (84.6%) ^a^	26 (70.3%) ^a, b^	< 0.001
Birthweight ≤ 1500 g	21 (45.7%) ^a, b^	25 (65.8%) ^a^	27 (67.5%) ^a^	5 (38.5%) ^b^	28 (75.7%) ^a, b^	0.02

Respondents could select more than 1 criteria. Superscript letters indicate groups significantly different within each criterion

*Respondents working across multiple levels of care. **Fisher-Freeman-Halton Exact Test

The frequency of DHM usage was variable, with almost 40% of HP reporting using DHM within their workplace daily or weekly, 28% fortnightly or monthly, and 28% rarely (Table 3). Almost half (48%) of all HP stated that they often would like to use DHM to feed their patients; however, they are unable to due to limited availability. DHM utilisation was 2.3 times more frequently available at district health boards located in the South Island than those in the North Island (87% vs. 37%, respectively, p = < 0.01, data not shown).

### Human milk donation expenses

Parents reported that costs associated with the HM donation arrangements (e.g., screening, pumping material, transport) were frequently covered by the donor (37.5%) or recipient parent (29%, Table 4). Of those who selected ‘other’ (15%), the majority of costs were shared between the donor and recipient parents, or partially covered with support from charities, healthcare system or a HM bank (data not shown).

HP reported that the associated expenses of HM donation (e.g., screening, pasteurisation, nutritional composition assessment) were most frequently covered by the healthcare system (39%). HP working under a district health board or community paediatric service frequently reported that expenses were covered by the healthcare system (44%), while those working privately or self-employed reported costs were covered by the donor and/or parent of the receiving infant (32%, data not shown).

### Experience and opinions with use of donor human milk

**Fig. 1:**
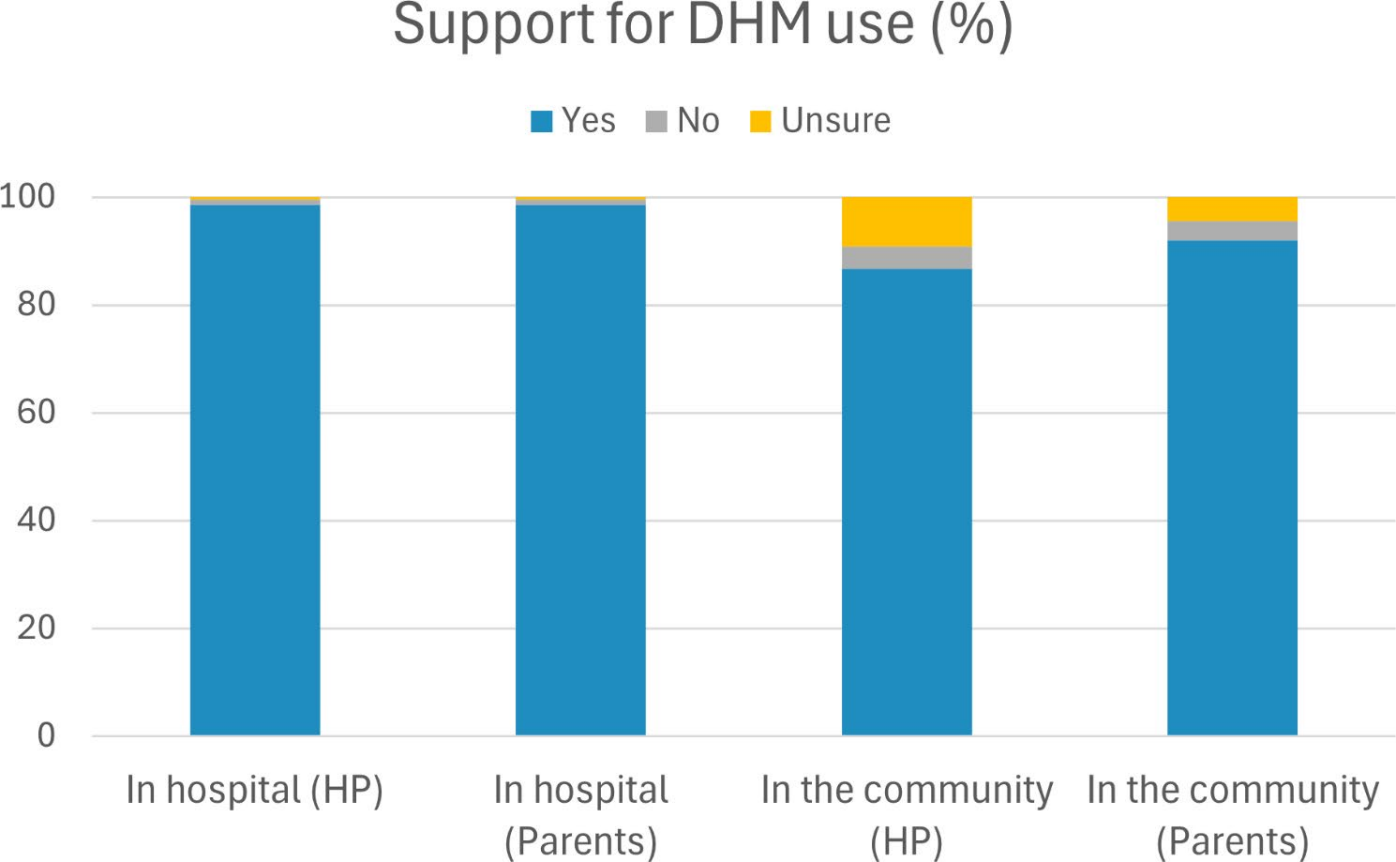
Overall parental (*n* = 454) and health professional (*n* = 218) support for the use of donor human milk in hospital and in the community (informal milk sharing). Figures are presented as percentage

Overall, almost all parents (98%) and HP (98%) supported the use of DHM in hospitals. Support for the use of DHM in the community was more common among parents (92%) than HP (87%), yet not statistically different (Fig. 1).

**Fig. 2:**
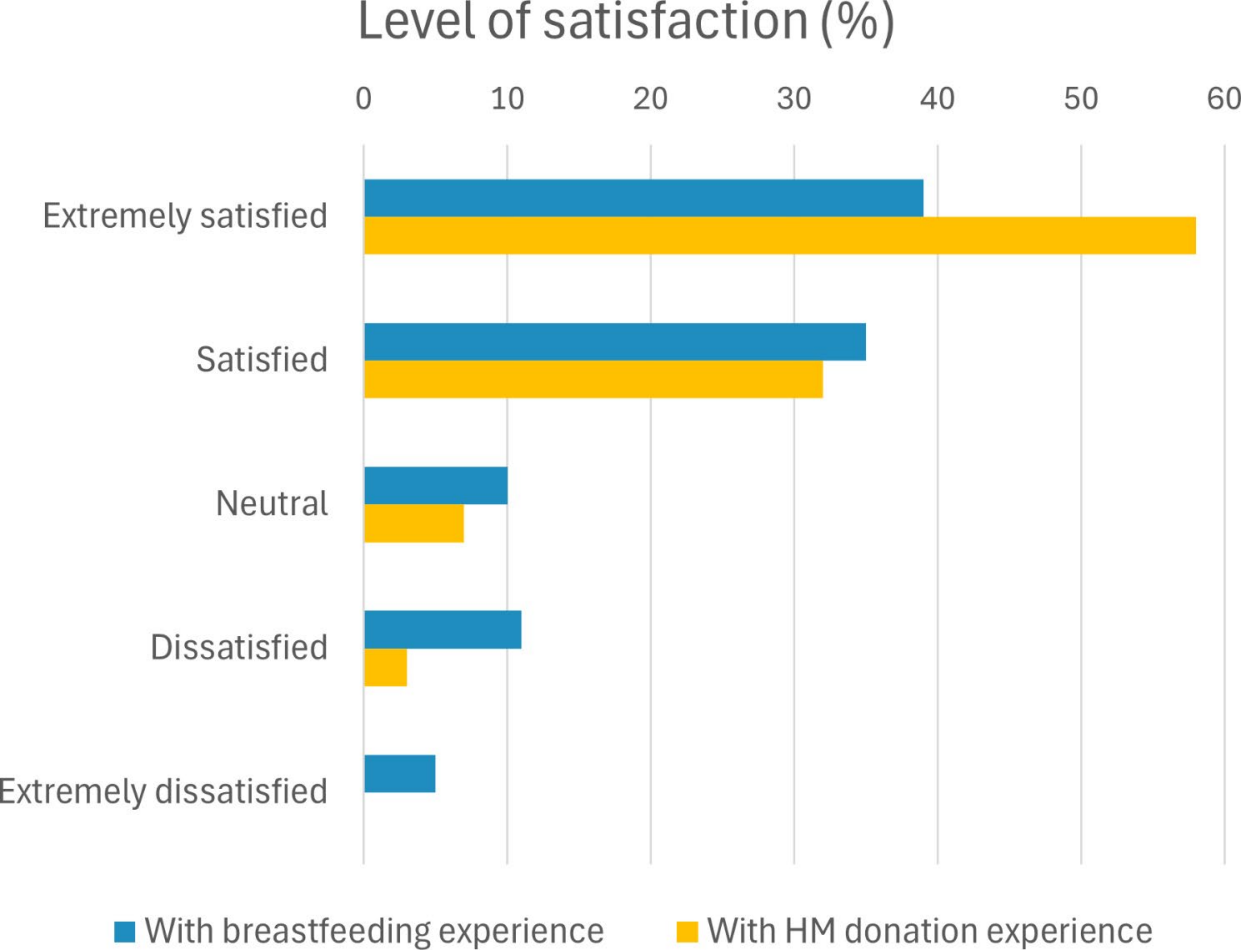
Level of satisfaction with breastfeeding experiences (parents, *n* = 494) and human milk donation (donors, *n* = 170). Figures are presented as percentage of responses

Most parents received some support from a lactation expert (e.g., lactation consultant, midwife, or lead maternity carer) regarding initiating or maintaining breastfeeding (85%, Table 2), with overall high levels of satisfaction with their breastfeeding experience (Fig. 2). However, some (25%) reported being neutral, dissatisfied or extremely dissatisfied with their breastfeeding experience, often as a result of their infant or themselves having difficulties breastfeeding (60%).

Most donors were satisfied with their HM donation experience (90%, Fig. 2). Among those who were dissatisfied or neutral with their HM donation experience (10%), some reported that a lack of structure made donating milk cumbersome and time-consuming. Some HM donors also reported feeling they did not receive adequate information or support to donate their milk effectively (*n* = 6) or having poor experiences due to feeling pressured to continue donating (*n* = 2), having their milk rejected by HM banks (*n* = 4) and the feeling the burden of obtaining HM donations and paying for resources (*n* = 2).

### Insights of human milk donation practices in New Zealand

**Fig. 3:**
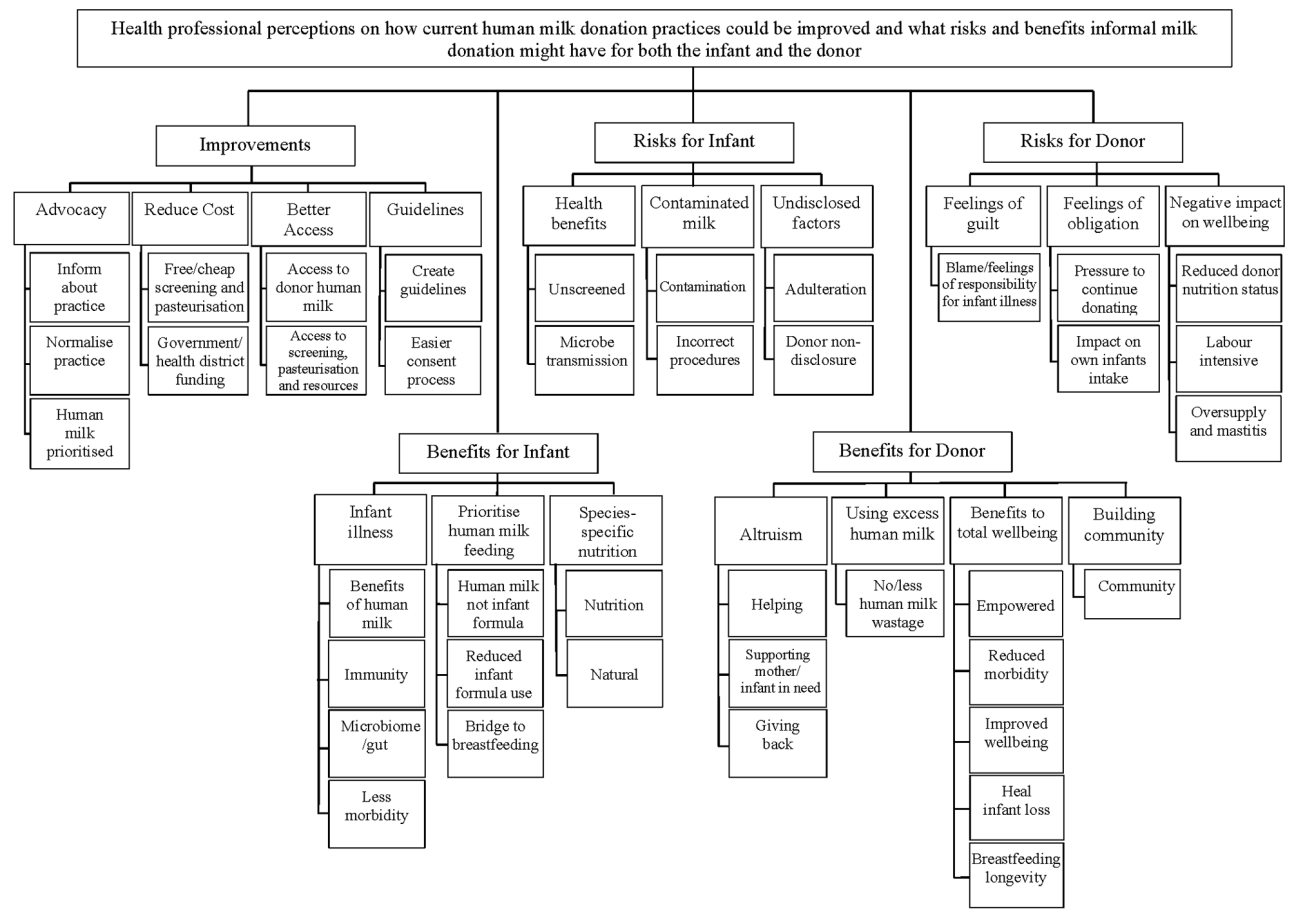
Codes and themes reflecting parents’ perceptions on current human milk donation practices and the potential risks and benefits of informal human milk donation for both infants and donors

**Fig. 4:**
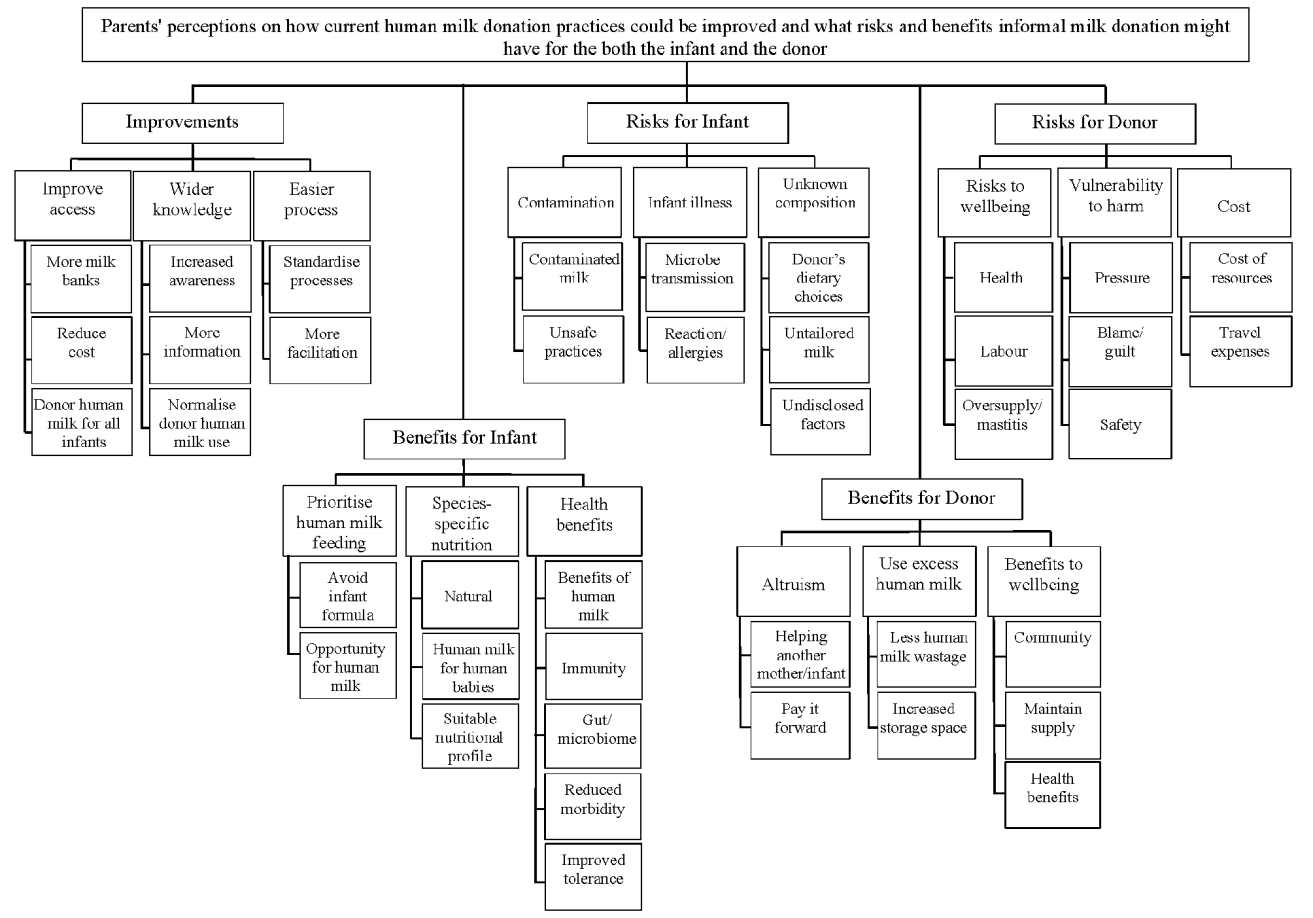
Codes and themes reflecting health professionals’ perceptions on current human milk donation practices and the potential risks and benefits of informal human milk donation for both infants and donors

The respondents were asked how they felt HM donation in NZ could be improved and what risks and benefits they perceived the practice might have for the donor and for the infant. Identified codes and themes are shown in Figs. 3 and 4.

### Improvements to current informal human milk donation practices

A total of 428 parents (86%) and 173 HP (72%) provided insights into how current informal HM donation practices could be improved. Four main themes emerged from the responses provided: (a) access; (b) wider knowledge; (c) reduce costs, and (d) guidelines, (Figs. 3 and 4).

- *Access*

Both parents and HP felt that equitable access to DHM for all infants, irrespective of age and health status, would improve current HM donation practices, improve safety and increase parents’ participation in the practice. Parents felt that access to community- and hospital-based HM banks may also provide a “*more structured arrangement*”, enabling a “*quick*” and “*efficient*” exchange of HM. Furthermore, some parents shared their experiences of feeling “*cut off*” from accessing DHM due to limited HM bank supply, leaving them with no choice but to feed with infant formula.

b) *Wider knowledge*

Many respondents highlighted the need for more “*awareness*”, “*information*”, “*education*”, “*advertising*” and “*encouragement*” for donating and receiving DHM. Some respondents stated they wished they had known of HM donation earlier to avoid feeding with infant formula. Antenatal classes or during the in-hospital postpartum period were suggested as places to provide awareness about HM donation. Additionally, respondents expressed their desire to see DHM normalised and made commonplace, taking priority over infant formula as a first-line option of supplementation of mothers’ milk.

c) *Reduce costs*

By using words such as “*available*” or “*accessible*”, HP identified that mitigation of costs would inevitably make DHM use more attainable. HP and parents agreed that reducing costs through funding for screening, pasteurisation, transport or more local DHM drop-off/pick-up locations may increase the safety and accessibility for families seeking DHM.

d) *Guidelines*

Survey respondents voiced the need for a more systematic process to donate and/or receive DHM. Parents felt that the current system of informal HM donation feels “*haphazard*” and “*clandestine*,” making a “*long and drawn-out process*” to exchange HM. Some HP reflected on the lack of standardised pathways for informal HM donation which subsequently influenced their ability to safely facilitate the use of donor HM. Both parents and HP felt that establishing guidelines for HM donation would be beneficial for “*safe*” and “*easy*” exchange of DHM.

### Potential benefits of donor human milk for the infant

A total of 497 parents (90.5%) and 193 HP (80%) provided their views about what are the perceived benefits of using DHM for the infant and three main themes emerged: (a) health benefits; (b) species-specific nutrition, and (c) prioritise HM feeding, (Figs. 3 and 4).

- *Health benefits*

Respondents commonly felt that informal milk sharing was beneficial to infant’s health, including a positive effect on infants’ microbiome/gut, immune system, and with the potential to reduce the risk of short- (necrotising enterocolitis, infection) and long- (diabetes, neurodevelopment, asthma, obesity, eczema) term morbidity compared to infant formula feeding.

b) *Prioritise human milk feeding*

Many parents highlighted that DHM provided an opportunity to feed the infant exclusively HM instead of infant formula, suggesting it was “*better alternative than formula*” and contained a wide range of components that infant formula “*will never be able to imitate*”. By using words such as “*choice*”, “*opportunity*”, “*option*”, “*alternative*” and “*preference*”, parents demonstrated their perceptions that DHM widens the potential feeding option for infants and can help some parents attain their goal of avoiding the “*use of*” or *“exposure to”* infant formula. Similarly, many HP felt that HM donation provided a gateway to HM feeding through “*helping*”, “*supporting*”, “*encouraging*” or “*promoting*” parents in their journey of breastfeeding establishment and continuation.

c) *Species-specific nutrition*

Respondents felt that DHM was a physiologically suitable feeding choice for infants, providing infants with “*natural*”, “*optimal*” or “*perfect*” “*species-specific*” nutrition.

### Potential risks of donor human milk for the infant

A total of 441 parents (89%) and 194 (81%) HP reflected on what could be the potential risks of current HM donation practices for the infant and three main themes emerged: (a) contamination; (b) infant illness; and (c) unknown composition, (Figs. 3 and 4).

- *Contamination*

Many parents identified that informally obtained DHM could cause harm to an infant as a result of poor hygiene and improper handling, storage, thawing, reheating and transport. Similarly, HP felt that informal HM donations may be tainted by products (drugs, medications, alcohol), poor handling processes (collection, storage, transit), or general lack of hygiene, all of which have the potential to cause harm to the infant. Improper processes, lack of safety information/guidelines and poor health literacy could further compromise safety of DHM.

b) *Infant illness*

Both parents and HP commonly identified transmission of pathogenic microbes from the donor to the ingesting infant as a risk of informal milk sharing. Some participants used words such as “*low*”, “*potential*”, “*possible*” or “*minimal*” as precursors to “*risk*” to emphasise that although there is a risk of microbiological transmission, the risk is not considerable. Respondents felt that the risk of infant illness was significantly lower if the milk and donor were adequately screened and the donated milk was pasteurised. Traces of allergens or unknown dietary factors were also described as having the potential to cause a harmful reaction for the ingesting infant.

c) *Unknown composition*

Both parents and HP expressed concern regarding the composition of informally obtained DHM and felt that some donors may not fully disclose their medical, lifestyle or serological background. Phrases such as “*undisclosed*”, “*unknown*”, “*not honest*” were used in combination with lifestyle and medical factors such as pharmaceuticals, recreational drugs, alcohol, or smoking. Furthermore, some parents reported that the DHM may contain antibodies inferior to those of the infants’ mother, diurnal changes, or have a nutritional profile incompatible with their infant’s age and nutritional needs.

### Potential benefits of human milk donation for the donor

A total of 267 HM donors (90%) and 190 (79%) HP provided insights into what could be potential benefits of HM donation for the donor. Three main themes emerged: (a) altruism; (b) using excess milk; and (c) benefits to total wellbeing, (Figs. 3 and 4).

- *Altruism*

Many donors discussed positive feelings associated with donating their milk to another parent and infant in need. Respondents used words such as “*satisfying*”, “*valuable*”, “*soul-warming*”, “*pride*”, “*helpful*”, “*supporting*”, “*fulfilment*” or “*amazing*” to emphasise the altruistic sentiments that came from donating milk. Some donors – especially those who had both donated and received DHM for their infant – also felt that HM donation was a way to give back to those who had previously supported them.

b) *Use excess human milk*

Respondents expressed that HM donation prevented excess milk from going to waste, and instead, could be valuable to other infants in need and mitigating feelings of guilt and reluctance when needing to discard their “*liquid gold*”.

c) *Benefits to total wellbeing*

Many physical, emotional, mental and social factors were reported to positively affect the donor’s wellbeing following HM donation. Respondents highlighted that donors’ physical health may improve by decreasing the risk of breast cancer, stimulating weight loss, relieving discomforts from engorgement and reduce morbidity (diabetes, lactational amenorrhoea, cancer and cardiovascular disease). Donors and HP also acknowledged the mental and emotional benefits of donating milk, with donors highlighting an increased sense of purpose, self-achievement, empowerment, altruism and social connections, such as developing community, friendships and sisterhood. Some HP further discussed the benefit that HM donation can have for parents who have lost their infant and on their grieving process.

### Potential risks of human milk donation for the donor

A total of 257 parents (86%) and 184 HP (77%) shared their views on what could be the potential risks of HM donation for the donor, with 32% of parents stating there were no or minimal risks for the donor. However, three main themes emerged following thematic analysis: (a) negative impact on donor wellbeing; (b) vulnerability to harm; and (c) cost, (Figs. 3 and 4).

- *Negative impact on donor wellbeing*

Parents and HP felt that HM donation could present risks to a donor’s mental and physical health, such as risk of mastitis, blocked milk ducts, dehydration, excessive weight loss, nutrient depletion, nipple trauma or hyperlactation resulting from increasing their milk supply to provide milk for another infant. Respondents also highlighted that such issues may subsequently affect the donor’s ability to breastfeed their own infant. One parent shared her experience of being diagnosed with pregnancy and lactation-associated osteoporosis attributable to hyperlactation, culminating in fractures. Furthermore, parents and HP recognised the effort, labour, cost and time required to pump extra milk.

b) *Vulnerability to harm*

Respondents also discussed how donors may be vulnerable to blame or feelings of responsibility if the recipient infant were to become sick after consuming their donated milk, especially via informal HM donation exchanges. Furthermore, parents and HP felt that donors may also be at risk of exploitation and pressure by the parent of the recipient infant, such as pressure to continue donating or to donate more milk than they are comfortable supplying, or that it can be difficult to say no to family or friends in need.

c) *Costs*

Some parents also highlighted the potential financial implications that informal HM donation may have for the donor, often attributable to serological screening, travel and resources such as a pump and milk bags, or even the additional financial burden if the recipient parent does not reimburse the donor for such costs.

## Discussion

Our findings demonstrate that informal milk sharing in NZ is common in hospitals and communities and both parents and HP are supportive of this practice. However, the results also indicate that current HM donation practices vary widely and are not equitable nationwide. Parents and HP feel there is a lack of awareness regarding HM donation in NZ and that, with greater knowledge, there would be improved involvement from both donors and parents of recipient infants, thereby increasing accessibility to DHM. Milk donation was discussed as a way of improving an infant’s health and avoiding unnecessary exposure to infant formula, but our survey also highlighted some concerns regarding potential risks for infants and donors and safety of using unpasteurised DHM for infant feeding. Establishing standardised guidelines for informal HM donation is required to mitigate potential risks and investing in HM banks will ensure equitable access to DHM for vulnerable infants.

The high rates of engagement with both formal and informal HM donation are in line with data from the first HM bank in NZ, located in Christchurch (South Island), which demonstrated that from 2014 to 2017 the number of HM donors and infants receiving DHM had increased by 240% and 130%, respectively [26]. Furthermore, a survey of nutrition practices among preterm infants in NZ and Australia Neonatal Intensive Care Units (NICU) indicated that unpasteurised DHM was provided in almost 40% of facilities, lower than reported in our study [11].

Our survey revealed a high prevalence of DHM use in NZ and particularly of informal milk donation (milk sharing), which was reported by 8 in 10 parents involved in this survey. Currently, there are only six active HM banks in NZ, which are spread between the North and South Islands and are a mixture of hospital-based (3) and community (3) HM banks. However, Auckland, which is the largest and most populated city, has no HM bank and is mostly reliant on informal (peer-to-peer) milk sharing. Studies in the US also suggest a high prevalence of mothers engaging in informal milk sharing as donors (12–69%) or recipients (7–44%), mostly occurring in the community and facilitated through the internet [27, 28]. Despite high rates of informal milk sharing in our survey and the literature [11, 29–32], there is an evident need for improved access to DHM and safe informal HM exchange, as without adequate screening and pasteurisation of donated milk, vulnerable infants may be at an increased risk of illness.

Our survey also identified a high proportion of informal milk sharing in clinical settings, possibly due to the limited available HM banks. A study based in Australia found that up to 75% of mothers of infants born moderate-late preterm (MLP) would have considered giving their infant pasteurised DHM from a HM bank during their infants’ hospitalisation, but it was unavailable to them [33]. Likewise, this study also found a proportion of mothers who wished that they had the option of providing DHM for their infants, but DHM was not available or unknown to them. Despite evident disparities in access, our findings highlight the high interest in DHM and increasing awareness of the potential benefits of HM feeding over infant formula.

An important finding from the HP survey is that DHM was commonly used for MLP and early term infants. Despite MLP infants accounting for the majority of preterm infants admitted to neonatal units in NZ and globally, there is a large gap in the current literature investigating the best nutritional approach and whether the use of pasteurised DHM in this population provides any health benefits as it does for very preterm and very low birthweight infants [13, 34]. Our findings mirror those reported by the Christchurch HM bank, whereby MLP infants represent a significant proportion of those who required supplementation of mothers’ milk [26]. While we did not ask about infants’ gestational age at birth, the majority of parents reported that their infant required some postnatal care (admission to NICU, Special Baby Care Unit or postnatal ward). Studies of NICUs and postnatal wards across the United States [35], Poland [36], China [37], Vietnam [38] and Taiwan [39] have also reported that DHM is often allocated to infants born between 32 and 38 weeks’ gestation or with a birthweight above 1500 g. Such findings may be because this population represents most infants requiring neonatal care [40–42]. In contrast, some studies of NICUs in Japan [43] and Ireland [44] indicate that DHM is most often utilised for very premature infants (< 32 weeks’ gestation) or infants with a birthweight below 1500 g, where the evidence of clinical benefit is clear [13, 45].

This survey found that in places with access to HM banks (i.e., Christchurch in the South Island), DHM was most often available for short periods to support mothers until hospital discharge and as a short-term bridge until their breastmilk supply was established, as previously reported in the literature [27, 46, 47]. In contrast, informal milk donations were often used for over four weeks, suggesting that some parents were using DHM as an ongoing supplement or substitute for their milk to avoid infant formula [28, 48], possibly indicating a lack of support for establishment and continuation of breastfeeding and a desire to avoid infant formula; as described in our qualitative responses and the literature [49–51].

International HM bank guidelines and informal milk sharing recommendations [52–57] emphasise the importance of adequate screening of the donor (particularly serological, medical and lifestyle screening), pasteurisation and microbiological testing of DHM prior to consumption among hospitalised infants [53, 55]. Pasteurisation is often undertaken by HM banks with specialised staff and equipment, which accounts for the bulk of costs in HM banking; however, informal HM donation places the choice of screening and pasteurisation with the donor and recipient mother [56], as observed in our study. Microbiological testing reported in our survey was low - likely because this type of screening is mostly performed by HM banks. Nevertheless, respondents recognised potential issues could be lessened with development and dissemination of informal HM donation guidelines.

Unscreened and unpasteurised milk may carry harmful pathogens, such as CMV, which can cause serious illness to an immature infant. Studies report that between 84 and 100% of mothers are positive for CMV but remain asymptomatic [58]. As such, one study found that 21% of HM samples anonymously purchased from a popular US informal milk sharing website were positive for CMV DNA [24]. However, it remains unclear whether such risks are significant for healthy and more mature infants (such as MLP and term infants) as it is for more vulnerable preterm infants. Furthermore, informal milk sharing presents the risk of exposure to contaminants such as drugs, chemicals or microbes introduced during handling, storage or transport [24, 58, 59] which were identified in our surveys, but could be mitigated by standardised practices and clear guidelines.

Most survey respondents reported screening the donor only for lifestyle and serological parameters. Compared to studies based in the US, where lifestyle and serological screening is undertaken by 5–72% and 3–27% of mothers [22, 60], respectively, NZ parents engaging in informal HM donation reported higher rates of serological screening of the donor. This may be because some NZ trusts/charities, such as Mother’s Milk NZ [61], provide partial funding for serological screening and informal milk sharing facilitated by a HP may be more likely to have serological screening of the donor requested prior to the exchange, which might be covered by the healthcare system.

This study provides some of the first insights into mothers and HP experiences and perceptions regarding both formal and informal HM donation practices in NZ, improving our understanding of the strengths and weaknesses of current HM donation practices in places where HM banks are not widely available. The information gathered in this study can inform future guidelines regarding informal HM donation, both within national and international context.

Surveys were distributed and circulated via online platforms, providing nationwide reach. However, participation was restricted to only those with access to social media, possibly over-representing the experiences and opinions of those with higher socioeconomic status and urban settings, as limited access to internet is associated with lower economic status and/or residing in remote areas in NZ [62]. Our finding could be susceptible to selection bias, as the respondents were not fully representative of the NZ birthing population, with higher participation of NZ/European in both the parents and HP surveys. Māori, the Indigenous people of NZ, currently represent 26% of the NZ birthing population [63]; however, only 6% and 8% of HP and parents, respectively, identified as Māori. With a lack of ethnic diversity, the survey findings may disproportionately reflect the viewpoints and practices of certain ethnic groups more likely to engage in HM donation, limiting the generalisability of the survey findings to the wider NZ population. Furthermore, parents of LGBTQIA + communities unable to breastfeed could potentially benefit from using DHM as they may be less likely to breastfeed their infants if hormonal induction of lactation is not feasible; however, HM donation practices of same-sex or foster parents was out of the scope of the current research and further investigation may be warranted. For these reasons, findings must be interpreted with caution as the external validity could be limited.

## Conclusion

Informal milk sharing in NZ is common and highly supported by parents and HP. However, limited structure, guidance and lack of standardised operations prevent equitable access to DHM. Establishing national and standardised guidelines for milk sharing is required to minimise the potential risks associated with informal HM donation. More support for HM banks in New Zealand is urgently needed to ensure all hospitalised vulnerable infants have access to DHM.

### Electronic supplementary material

Below is the link to the electronic supplementary material.

**Supplementary Material 1**: Electronic surveys for parents and health professionals containing open- (free text) and closed- (multiple choice) questions

## Data Availability

The datasets generated and/or analysed during the current study can be made available through reasonable request submitted to the corresponding author upon review of the proposed purpose and if consistent with the ethical approval obtained.

## Abbreviations

DHM: Donor human milk
HM: Human milk
HP: Health professionals
MLP: Moderate-late preterm
NICU: Neonatal Intensive Care Unit
NZ: New Zealand
