# Supplementary material for: Formal and informal human milk donation in New Zealand: a mixed-method national survey
Source: Int Breastfeed J. 2024 Sep 2;19:61. doi: 10.1186/s13006-024-00667-4 (PMC11370131; doi:10.1186/s13006-024-00667-4)
Supplement: Supplementary file 1 — Supplementary Material 1: Electronic surveys for parents and health professionals containing open- (free text) and closed- (multiple choice) questions [file 13006_2024_667_MOESM1_ESM.pdf]

Human milk donation in Aotearoa New Zealand: Parents' Survey

Q1. Please select your age group.

- ◯ <18 (1)
- ◯ 18-29 (2)
- ◯ 30-39 (3)
- ◯ >40 (4)
- ◯ Prefer not to disclose (8)

Q2. Which ethnic group do you belong to? (Select all which apply to you)

- ▢ New Zealand European (1)
- ▢ Māori (2)
- ▢ Samoan (3)
- ▢ Cook Islands Māori (4)
- ▢ Tongan (5)
- ▢ Niuean (6)
- ▢ Chinese (7)
- ▢ Indian (8)
- ▢ Other (Please state, eg, Dutch, Japanese, Tokelauan) (9) ____

Q3. Where was your baby/pēpi born?

▼ Select the region from the options below.

Q4. In which type of facility did you give birth?

- ◯ Hospital (1)
- ◯ Birthing centre/ maternity unit (2)
- ◯ Home birth (3)
- ◯ Other (4) ____

Q5. Did your baby/pēpi receive postnatal care?

- ◯ Yes - in the postnatal ward (1)
- ◯ Yes - under the Special Care Baby Unit (2)
- ◯ Yes - under the Neonatal Intensive Care Unit (3)
- ◯ No - my baby/pēpi did not require postnatal hospital care (4)
- ◯ Other: (5) ____

*

Q6. How many children/tamariki do you have?

____

*

Q7. How many babies/pēpi have you breastfed/whāngote (past and present)?

____

Q8. What is the age of your youngest child/pōtiki?

- ◯ 0-6 months old (1)
- ◯ 7-12 months old (2)
- ◯ 1-2 years old (3)
- ◯ ≥2 years old (4)

Q9. What is the sex of your youngest child/pōtiki?

- ◯ Boy (1)
- ◯ Girl (2)
- ◯ Twins of different sex (4)
- ◯ Prefer not to disclose (5)

Q10. How have you been involved with breast milk/waiū donation?

- ◯ I have donated/shared my own milk/waiū to help someone else's mother/baby (māmā/pēpi) (1)
- ◯ My baby/pēpi received donated breast milk/waiū **during their hospital stay** (2)
- ◯ My baby/pēpi received donated breast milk/waiū **after discharge from hospital** (3)
- ◯ I have **both** donated my own milk/waiū and my baby/pēpi received donated breast milk/waiū (7)
- ◯ I have directly breastfed/whāngote someone else's baby/pēpi **or** my baby/pēpi was directly breastfed/whāngote by another mother/māmā (10)
- ◯ I wish I had the option to be involved with breast milk/waiū donation but it was not available to me (8)

Q11. What mode of feeding did your baby/pēpi receive in their first six months of life?

- ◯ My baby/pēpi was exclusively fed my own milk/waiū (from the breast or expressed milk) (1)
- ◯ My baby/pēpi was partially fed my own milk/waiū and infant formula (2)
- ◯ My baby/pēpi was partially fed my own milk/waiū and donated breast milk (3)
- ◯ My baby/pēpi was partially fed donated breast milk/waiū and infant formula (4)
- ◯ My baby/pēpi was exclusively fed infant formula (5)
- ◯ My baby/pēpi was exclusively fed donated breast milk/waiū (6)
- ◯ Other: (7) ____

Q40 Q12. Did you receive any support from a health professional to initiate or maintain breastfeeding/whāngai ū? (e.g. lactation consultant, midwife, lead maternity carer)

- ◯ Yes (1)
- ◯ No (2)

Q13. How satisfied were you with your breastfeeding/whāngai ū experience?

- ◯ Extremely satisfied (1)
- ◯ Satisfied (2)
- ◯ Neither satisfied nor dissatisfied (3)
- ◯ Dissatisfied (4)
- ◯ Extremely dissatisfied (5)

Display This Question:

If Q13. How satisfied were you with your breastfeeding/whāngai ū experience? = Neither satisfied nor dissatisfied

Or Q13. How satisfied were you with your breastfeeding/whāngai ū experience? = Dissatisfied

Or Q13. How satisfied were you with your breastfeeding/whāngai ū experience? = Extremely dissatisfied

Q14. For what reason(s) did you select your answer to the above question? Select all that apply.

- ☐ My baby/pēpi had difficulty breastfeeding/whāngai ū (1)
- ☐ I did not feel emotionally/physically supported enough to continue breastfeeding/whāngai ū (2)
- ☐ My baby/pēpi did not gain enough weight (3)
- ☐ I had difficulty breastfeeding/whāngai ū (e.g. pain, mastitis, cracked and/or bleeding nipples) (4)
- ☐ I no longer wished to breastfeed/whāngote (5)
- ☐ Other: (6) ____

Display This Question:

If Q10. How have you been involved with breast milk/waiū donation? = My baby/pēpi received donated breast milk/waiū <u>after discharge from hospital</u>

Or Q10. How have you been involved with breast milk/waiū donation? = I have <u>both</u> donated my own milk/waiū and my baby/pēpi received donated breast milk/waiū

Or Q10. How have you been involved with breast milk/waiū donation? = My baby/pēpi received donated breast milk/waiū <u>during their hospital stay</u>

Q15. What were your reasons/motivations to seek donated breast milk/waiū to supplement your own milk/waiū?

____

____

____

Display This Question:

If Q10. How have you been involved with breast milk/waiū donation? = I have donated/shared my own milk/waiū to help someone else's mother/baby (māmā/pēpi)

Or Q10. How have you been involved with breast milk/waiū donation? = I have <u>both</u> donated my own milk/waiū and my baby/pēpi received donated breast milk/waiū

Q16. What were your reasons/motivations to donate your milk/waiū?

Display This Question:

If Q10. How have you been involved with breast milk/waiū donation? = I have donated/shared my own milk/waiū to help someone else's mother/baby (māmā/pēpi)

Or Q10. How have you been involved with breast milk/waiū donation? = My baby/pēpi received donated breast milk/waiū <u>during their hospital stay</u>

Or Q10. How have you been involved with breast milk/waiū donation? = My baby/pēpi received donated breast milk/waiū <u>after discharge from hospital</u>

Or Q10. How have you been involved with breast milk/waiū donation? = I have <u>both</u> donated my own milk/waiū and my baby/pēpi received donated breast milk/waiū

Q17. How was the breast milk/waiū donation arrangement facilitated? Select all that apply.

- ☐ Facilitated through the hospital health professionals (e.g. lactation consultant, midwife, lead maternity carer) (1)
- ☐ Facilitated through a breast milk bank (2)
- ☐ Organised between individuals (family/friends/internet/social media) (3)
- ☐ Facilitated through charities/organisations (e.g. Plunket, La Leche League) (4)
- ☐ Other (5) ____

Display This Question:

If Q10. How have you been involved with breast milk/waiū donation? = My baby/pēpi received donated breast milk/waiū <u>after discharge from hospital</u>

Or Q10. How have you been involved with breast milk/waiū donation? = My baby/pēpi received donated breast milk/waiū <u>during their hospital stay</u>

Or Q10. How have you been involved with breast milk/waiū donation? = I have <u>both</u> donated my own milk/waiū and my baby/pēpi received donated breast milk/waiū

Q18. For how long did you use donated breast milk/waiū?

- ○ <1 week (1)
- ○ 1-2 weeks (2)
- ○ 3-4 weeks (3)
- ○ 4+ weeks (4)

Display This Question:

If Q10. How have you been involved with breast milk/waiū donation? = My baby/pēpi received donated breast milk/waiū <u>after discharge from hospital</u>

Or Q10. How have you been involved with breast milk/waiū donation? = My baby/pēpi received donated breast milk/waiū <u>during their hospital stay</u>

Or Q10. How have you been involved with breast milk/waiū donation? = I have <u>both</u> donated my own milk/waiū and my baby/pēpi received donated breast milk/waiū

Q19. How often was donated breast milk/waiū used to feed your baby/pēpi?

- ◯ I used donated breast milk/waiū multiple times per day (1)
- ◯ I used donated breast milk/waiū once daily (2)
- ◯ I used donated breast milk/waiū weekly (3)
- ◯ I used donated breast milk/waiū fortnightly (4)
- ◯ I infrequently used donated breast milk/waiū (less than fortnightly) (5)

Display This Question:

If Q10. How have you been involved with breast milk/waiū donation? = I have donated/shared my own milk/waiū to help someone else's mother/baby (māmā/pēpi)

Or Q10. How have you been involved with breast milk/waiū donation? = I have <u>both</u> donated my own milk/waiū and my baby/pēpi received donated breast milk/waiū

Q20. I have donated my milk/waiū to:

- ◯ 1 baby/pēpi (1)
- ◯ 2 babies/pēpi (2)
- ◯ 3 babies/pēpi (3)
- ◯ 4 babies/pēpi (4)
- ◯ 4+ babies/pēpi (5)
- ◯ I am unsure how many babies/pēpi have received my donated milk/waiū (6)

Display This Question:

If Q10. How have you been involved with breast milk/waiū donation? = I have donated/shared my own milk/waiū to help someone else's mother/baby (māmā/pēpi)

Or Q10. How have you been involved with breast milk/waiū donation? = I have <u>both</u> donated my own milk/waiū and my baby/pēpi received donated breast milk/waiū

Q21. How frequent were your milk/waiū donations?

- ◯ Daily (1)
- ◯ Weekly (2)
- ◯ Monthly (3)
- ◯ One-off donation (4)

Display This Question:

If Q10. How have you been involved with breast milk/waiū donation? = I have donated/shared my own milk/waiū to help someone else's mother/baby (māmā/pēpi)

Or Q10. How have you been involved with breast milk/waiū donation? = I have <u>both</u> donated my own milk/waiū and my baby/pēpi received donated breast milk/waiū

Q22. For how long did you donate your milk/waiū?

- ◯ <1 month (1)
- ◯ 1-2 months (2)
- ◯ 3-4 months (3)
- ◯ 5-6 months (4)
- ◯ 6+ months (5)

Display This Question:

If Q10. How have you been involved with breast milk/waiū donation? = I have donated/shared my own milk/waiū to help someone else's mother/baby (māmā/pēpi)

Or Q10. How have you been involved with breast milk/waiū donation? = I have <u>both</u> donated my own milk/waiū and my baby/pēpi received donated breast milk/waiū

Q23. How satisfied were you with your milk/waiū donation experience?

- ◯ Extremely satisfied (1)
- ◯ Satisfied (2)
- ◯ Neither satisfied nor dissatisfied (3)
- ◯ Dissatisfied (4)
- ◯ Extremely dissatisfied (5)

Display This Question:

If Q23. How satisfied were you with your milk/waiū donation experience? = Neither satisfied nor dissatisfied

Or Q23. How satisfied were you with your milk/waiū donation experience? = Dissatisfied

Or Q23. How satisfied were you with your milk/waiū donation experience? = Extremely dissatisfied

Q24. For what reason(s) did you select your answer to the above question? (e.g. time-consuming, too much responsibility)

____

____

____

____

____

Display This Question:

If Q10. How have you been involved with breast milk/waiū donation? = I have donated/shared my own milk/waiū to help someone else's mother/baby (māmā/pēpi)

Or Q10. How have you been involved with breast milk/waiū donation? = My baby/pēpi received donated breast milk/waiū <u>during their hospital stay</u>

Or Q10. How have you been involved with breast milk/waiū donation? = My baby/pēpi received donated breast milk/waiū <u>after discharge from hospital</u>

Or Q10. How have you been involved with breast milk/waiū donation? = I have <u>both</u> donated my own milk/waiū and my baby/pēpi received donated breast milk/waiū

Q25. Which of the following screening processes occurred prior to the donated breast milk/waiū arrangement? Select all that apply.

- ☐ Serological (e.g. blood testing for antibodies against HIV, CMV, Hepatitis C or B and syphilis) (1)
- ☐ Microbiological (e.g. bacterial contamination testing) (2)
- ☐ Lifestyle (e.g. smoking status, medication, drug and alcohol intake) (3)
- ☐ No screening arrangements were undertaken prior to my donated breast milk/waiū experience (4)
- ☐ I am unsure whether screening arrangements were undertaken prior to my donated breast milk/waiū experience (5)

Display This Question:

If Q10. How have you been involved with breast milk/waiū donation? = I have donated/shared my own milk/waiū to help someone else's mother/baby (māmā/pēpi)

Or Q10. How have you been involved with breast milk/waiū donation? = My baby/pēpi received donated breast milk/waiū <u>during their hospital stay</u>

Or Q10. How have you been involved with breast milk/waiū donation? = My baby/pēpi received donated breast milk/waiū <u>after discharge from hospital</u>

Or Q10. How have you been involved with breast milk/waiū donation? = I have <u>both</u> donated my own milk/waiū and my baby/pēpi received donated breast milk/waiū

Q26. Was the donated breast milk/waiū pasteurised (flash-heated) before being given to the baby/pēpi?

(pasteurisation: the process of heating breast milk/waiū to 62.5 °C for 30 minutes prior to being fed to the baby/pēpi to kill potentially harmful microbes).

- ◯ Yes - the donated milk/waiū was pasteurised by the donor or receiving mother/māmā (1)
- ◯ Yes - the donated milk/waiū was pasteurised through the milk bank/hospital facilities (3)
- ◯ No - the donated milk/waiū remained unpasteurised (5)
- ◯ I don't know (6)

Display This Question:

If Q26. Was the donated breast milk/waiū pasteurised (flash-heated) before being given to the baby/p... = Yes - the donated milk/waiū was pasteurised by the donor or receiving mother/māmā

Q27. Please describe the pasteurisation process undertaken, if known.

Display This Question:

If Q10. How have you been involved with breast milk/waiū donation? = I have donated/shared my own milk/waiū to help someone else's mother/baby (māmā/pēpi)

Or Q10. How have you been involved with breast milk/waiū donation? = I have <u>both</u> donated my own milk/waiū and my baby/pēpi received donated breast milk/waiū

Q28. Did you make any dietary changes in order to donate your milk/waiū?

- ◯ No, I followed my normal diet (1)
- ◯ Yes, I changed some components of my diet, including: (2)

Q29. Were there any expenses associated with the breast milk/waiū donation arrangement? (e.g. screening, pasteurisation, transport)

- ◯ Yes (1) ____
- ◯ No (2)

Display This Question:

If Q29. Were there any expenses associated with the breast milk/waiū donation arrangement? (e.g. scr... = Yes

Q30. Who covered the costs associated with the breast milk/waiū donation arrangement?

- ◯ Associated costs were covered by the healthcare system (e.g. serological screening) (1)
- ◯ Associated costs were covered by the breast milk/waiū donor (2)
- ◯ Associated costs were covered by the mother/māmā of the receiving baby/pēpi (3)
- ◯ Associated costs were covered by health insurance (4)
- ◯ I don't know (7)
- ◯ Other: (5) ____

Display This Question:

If Q10. How have you been involved with breast milk/waiū donation? = I have directly breastfed/whāngote someone else's baby/pēpi <u>or</u> my baby/pēpi was directly breastfed/whāngote by another mother/māmā

Q31. How was your shared breastfeeding/whāngai ū experience? (e.g. your reasons or motivations)

____

Q32. Do you support the use of donor breast milk/waiū banks in hospitals?

- ◯ Yes (1)
- ◯ No (2)
- ◯ I don't know (4)

Q33. Do you support the use of informal breast milk/waiū donation arrangements between mothers/whāea?

- ◯ Yes (1)
- ◯ No (2)
- ◯ I don't know (3)

Q34. How could current breast milk/waiū donation arrangements be improved?

____

____

____

____

____

Q35. What do you think are the **benefits** of using donor milk/waiū for the **baby/pēpi**?

____

____

____

____

____

Q36. What do you think are the **risks** of using donor milk/waiū for the **baby/pēpi**?

____

____

____

____

____

Display This Question:

If Q10. How have you been involved with breast milk/waiū donation? = I have donated/shared my own milk/waiū to help someone else's mother/baby (māmā/pēpi)

Or Q10. How have you been involved with breast milk/waiū donation? = I have <u>both</u> donated my own milk/waiū and my baby/pēpi received donated breast milk/waiū

Q37. What do you think are the **benefits** of donating milk/waiū for the **donor**?

____

____

____

____

____

Display This Question:

If Q10. How have you been involved with breast milk/waiū donation? = I have donated/shared my own milk/waiū to help someone else's mother/baby (māmā/pēpi)

Or Q10. How have you been involved with breast milk/waiū donation? = I have <u>both</u> donated my own milk/waiū and my baby/pēpi received donated breast milk/waiū

Q38. What do you think are the **risks** of donating milk/waiū for the **donor**?

____

____

____

____

____

End of Survey

Human milk donation in Aotearoa New Zealand: Health Professionals' Survey

Q1. What is your profession? Select all that apply.

- ☐ Neonatologist (1)
- ☐ Dietitian (2)
- ☐ Lactation Consultant (3)
- ☐ Midwife (4)
- ☐ Nurse (5)
- ☐ Lead Maternity Carer (7)
- ☐ Paediatrician (8)
- ☐ Other: (6) ____

Q2. Which organisation do you work for?

- ○ District Health Board (1)
- ○ Plunket (3)
- ○ Private Care (7)
- ○ Self-employed (8)
- ○ Non-governmental Organisations/Charities/Trusts (e.g. La Leche League, Mothers Milk NZ Charitable Trust, Human Milk 4 Human Babies) (2)
- ○ Other: (9) ____

Display This Question:

If Q2. Which organisation do you work for? = District Health Board

Q3. Which District Health Board do you work for?

▼ Select from the options listed below

Q4. Which level of healthcare do you operate under? Select all that apply.

- ▢ Primary (professional health care provider in the community, e.g. practice nurse, GP, LMC) (1)
- ▢ Secondary (specialist level care) (2)
- ▢ Tertiary (specialist care for hospitalised patients) (3)
- ▢ Other (i.e. community based) (4)

Q5. How many years have you worked with newborns/pēpi in neonatal health?

- ◯ 0-5 (1)
- ◯ 6-10 (2)
- ◯ 11-15 (3)
- ◯ ≥ 15 (4)

Q6. What gender do you identify as?

- ○ Male (1)
- ○ Female (2)
- ○ Non-binary (3)
- ○ Prefer not to disclose (4)

Q7.Which ethnic group do you belong to? (Select all which apply to you)

- ☐ New Zealand European (1)
- ☐ Māori (2)
- ☐ Samoan (3)
- ☐ Cook Islands Māori (4)
- ☐ Tongan (5)
- ☐ Niuean (6)
- ☐ Chinese (7)
- ☐ Indian (8)
- ☐ Other (Please state, eg, Dutch, Japanese, Tokelauan) (9) ____
- ☐ Prefer not to disclose (10)

Q8. Please select your age group.

- ○ 18-29 (1)
- ○ 30-39 (2)
- ○ 40-49 (3)
- ○ ≥ 50 (4)
- ○ Prefer not to disclose (7)

Q9. Is donated human milk/waiū available within your workplace? Select all that apply.

- ☐ Yes - Facilitated through the hospital staff (e.g. lactation consultants, midwifes, nurses) (1)
- ☐ Yes - Facilitated through a human milk/waiū bank (2)
- ☐ Yes - Organised between individuals (whānau/friends/internet/social media) (3)
- ☐ No (4)

Skip To: If Q9. Is donated human milk/waiū available within your workplace? Select all that apply. = No

---

Q10. Which babies/pēpi usually receive donated human milk/waiū in your workplace? Select all that apply.

- ☐ Extremely preterm (less than 28 weeks) (1)
- ☐ Very preterm (28 to 32 weeks) (2)
- ☐ Moderate to late preterm (32 to 37 weeks) (3)
- ☐ Early term (37 to 38 weeks) (4)
- ☐ Full term (39 to 40 weeks) (5)
- ☐ Late term (41 to 42 weeks) (6)
- ☐ Post term (After 42 weeks) (7)
- ☐ Extremely low birth weight ((8)
- ☐ Very low birth weight ((9)
- ☐ Low birth weight ((10)

Q11. How often is donated human milk/waiū used within your workplace?

- ○ Often (e.g. daily/weekly) (4)
- ○ Sometimes (e.g. fortnightly/monthly) (3)
- ○ Rarely (e.g. quarterly/annually) (2)
- ○ Never (5)
- ○ I don't know (6)

Q12. How often would your workplace like to use donated human milk/waiū but cannot due to limited availability?

- Often (e.g. daily/weekly) (3)
- Sometimes (e.g. fortnightly/monthly) (2)
- Rarely (e.g. quarterly/annually) (1)
- Never (4)
- I don't know (5)

Q13. Are there any guidelines, protocols and/or policies available at your workplace related to informal milk/waiū donation and/or use of donor milk/waiū?

- Yes (1)
- No (2)
- I don't know (3)

Display This Question:

If Q13. Are there any guidelines, protocols and/or policies available at your workplace related to i... = Yes

Q14. Are you able to share these guidelines with us?

- Yes (1)
- No (2)

Display This Question:

If Q14. Are you able to share these guidelines with us? = Yes

Q35 Please upload any guidelines, protocols and/or policies available at your workplace related to informal human milk donation and/or use of donor milk:

Q15. Is the use of donated human milk/waiū documented within the clinical notes (e.g. observation charts, fluid balance charts)?

- ○ Yes (1)
- ○ No (2)
- ○ I don't know (3)

Display This Question:

If Q9. Is donated human milk/waiū available within your workplace? Select all that apply. = Yes - Facilitated through the hospital staff (e.g. lactation consultants, midwifes, nurses)

Or Q9. Is donated human milk/waiū available within your workplace? Select all that apply. = Yes - Facilitated through a human milk/waiū bank

Or Q9. Is donated human milk/waiū available within your workplace? Select all that apply. = Yes - Organised between individuals (whānau/friends/internet/social media)

Q33 Q16. Is informed consent from the mother/māmā required in order to use donor human milk/waiū to feed the baby/pēpi?

- ○ Yes (1)
- ○ No (2)
- ○ I don't know (4)

Q17. What type of screening process is usually undertaken on the donor and/or their donated human milk/waiū? Select all that apply.

- ☐ Serological (e.g. blood testing for antibodies against HIV, CMV, Hepatitis C or B and syphilis) (1)
- ☐ Microbiological (e.g. bacterial contamination testing) (2)
- ☐ Lifestyle (e.g. smoking status, medication, drug and alcohol intake) (3)
- ☐ The donor and/or the donated human milk/waiū are not screened (4)
- ☐ I don't know (5)

Q18. Is the nutritional composition of the donated human milk/waiū assessed?

- ○ Yes (1)
- ○ No (2)
- ○ I don't know (3)

Q19. Is the donated human milk/waiū pasteurised prior to the baby's/pēpi consumption?
(pasteurisation: the process of heating human milk/waiū to 62.5 °C for 30 minutes prior to being fed to the baby/pēpi to kill potentially harmful microbes).

- ○ Yes (1)
- ○ No (2)
- ○ I don't know (3)

Display This Question:

If Q19. Is the donated human milk/waiū pasteurised prior to the baby's/pēpi consumption? (pasteurisa... = Yes

Q20. Please describe the milk/waiū pasteurisation process used within your workplace.

____

____

____

____

____

Q21. How are the associated expenses (e.g. screening, pasteurisation, nutritional composition assessment) of donated human milk/waiū paid?

- ◯ Associated costs are covered by the health care system (1)
- ◯ Associated costs are covered by the individual (donor and/or mother/māmā of the receiving baby/pēpi) (2)
- ◯ Associated costs are covered by charitable organisations (3)
- ◯ Other: (4) ____
- ◯ I don't know (5)

Q22. In instances where donated human milk/waiū has not been completely used, what is done with the excess milk/waiū? Select all that apply.

- ☐ The excess milk/waiū is disposed of (1)
- ☐ The excess milk/waiū is stored frozen and used at a later date (2)
- ☐ The excess milk/waiū is offered to a low priority baby/pēpi (e.g. stable or healthy baby/pēpi) (3)
- ☐ The excess milk/waiū is used for research (5)
- ☐ I don't know (7)
- ☐ Other: (6) ____

Q23. Which category do you think donated human milk/waiū should fall under?

- ○ As a nutritional product prescribed by the medical team (equal to infant formula) (1)
- ○ As a nutritional supplement prescribed by the medical team (equal to vitamins and minerals) (2)
- ○ As medicine prescribed by the medical team (equal to pharmacological drugs) (3)

Page Break

Q24. Do you support the use of human donor milk/waiū banks in hospitals?

- ◯ Yes (1)
- ◯ No (2)
- ◯ I don't know (4)

Q25. Do you support the use of informal milk/waiū sharing in the community?

- ◯ Yes (1)
- ◯ No (2)
- ◯ I don't know (4)

Q26. How could current practices regarding informal milk/waiū donation be improved?

____

____

____

____

____

Q27. What do you think are the **benefits** of informal milk/waiū sharing for the **baby/pēpi**?

____

____

____

____

Q28. What do you think are the **risks** of informal milk/waiū sharing for the **baby/pēpi**?

Q29. What do you think are the **benefits** of informal milk/waiū sharing for the **donor**?

Q30. What do you think are the **risks** of informal milk/waiū sharing for the **donor**?

End of Survey
